# The imprisonment-extremism nexus: Continuity and change in activism and radicalism intentions in a longitudinal study of prisoner reentry

**DOI:** 10.1371/journal.pone.0242910

**Published:** 2020-11-30

**Authors:** Scott H. Decker, David C. Pyrooz

**Affiliations:** 1 School of Criminology and Criminal Justice, Arizona State University, Phoenix, AZ, United States of America; 2 Department of Sociology, University of Colorado Boulder, Boulder, CO, United States of America; Geneva University Hospitals, SWITZERLAND

## Abstract

There is considerable speculation that prisons are a breeding ground for radicalization. These concerns take on added significance in the era of mass incarceration in the United States, where 1.5 million people are held in state or federal prisons and around 600,000 people are released from prison annually. Prior research relies primarily on the speculation of prison officials, media representations, and/or cross-sectional designs to understand the imprisonment-extremism nexus. We develop a tripartite theoretical model to examine continuity and change in activism and radicalism intentions upon leaving prison. We test these models using data from a large probability sample of prisoners (*N* = 802) in Texas interviewed in the week preceding their release from prison and then reinterviewed 10 months later using a validated scale of activism and radicalism intentions. We arrive at three primary conclusions. First, levels of activism decline upon reentry to the community (*d* = -0.30, *p* < .01), while levels of radicalism largely remain unchanged (*d* = -0.08, *p* = .28). What is learned and practiced in prison appears to quickly lose its vitality on the street. Second, salient groups and organizations fell in importance after leaving prison, including country, race/ethnicity, and religion, suggesting former prisoners are occupied by other endeavors. Finally, while we identify few correlates of changes in extremist intentions, higher levels of legal cynicism in prison were associated with increases in both activism and radicalism intentions after release from prison. Efforts designed to improve legal orientations could lessen intentions to support non-violent and violent extremist actions. These results point to an imprisonment-extremism nexus that is diminished largely by the realities of prisoner reentry.

## Introduction

Total institutions, such as military camps, cloisters, boarding schools, and—the focus of our study—prisons, cut off individuals from the outside world, closely regulate behavior, impose strict regimens, and force their residents to associate almost exclusively with others like themselves [1]. Individuals enter total institutions with a set of normative beliefs that may be changed by long, intense periods of confinement, then changed yet again upon release to a less regulated social system outside the institution. The impact of such institutions can condition the attitudes and behavior of individuals well after they have left. Prisons are an archetypal total institution [1–4]. Understanding the impact of transitions into and out of prison is important for documenting individual changes in attitudes, behavior, and associations [5–9]. This is especially the case for release from prison back to the community, a transition experienced by 600,000 people in the United States each year [10]. Because of the magnitude of this transition, prisons have taken on greater significance in the era of mass incarceration [11–14], particularly with respect to violent extremism.

Although there is much speculation about the imprisonment-extremism nexus, there is little systematic analysis of this relationship. Much of the work in this area is journalistic [15] or based on “expert” opinion [16]. The impact of imprisonment on extremist attitudes and behaviors is plausible, as researchers have pointed to the incarceration histories of many violent extremists [17, 18]. Still, there is limited data on these issues, mostly because access to prisons in general is highly restricted, with much less access to prisoners who have extremist tendencies. Many analyses substitute the perceptions of correctional officers or staff for direct measurement of the attitudes or behaviors of prisoners. Other analyses note the plausibility of extremism among prisoners [19, 20], although without explicit observation or measurement of these concepts among individuals confined to prison. There is some cross-sectional research on this issue, but a one-time snapshot of current or former prisoners prohibits examining continuity and change in such associations, behaviors, and beliefs, nor does it account for the time order of imprisonment [21, 22]. Despite multiple calls for more systematic research on imprisonment and extremism, the research community has had little to offer on this topic despite its importance [21, 23–25]. And little as we understand the context of extremism in prison, we know even less about what happens when individuals leave prison. Thus, there are concerns both about what occurs in prison as well as what happens to these individuals when they return to the street. Extremist orientations may increase, remain the same, or decline with reentry to society; continuity *and* change have important implications for prison, reentry, recidivism, and extremism.

This study examines the imprisonment-extremism nexus in the context of a longitudinal study of prisoner reentry. We begin by offering a tripartite theoretical framework for continuity and change in extremist orientations after release from prison. Consistent with Moskalenko and McCauley [26], we differentiate between willingness to take action on behalf of a cause or a group into legal and illegal forms. *Activism* refers to legal and non-violent forms of political mobilization in support of a cause or a group, while *radicalism* refers to illegal and violent forms of political mobilization—affinity towards and investment in a cause or group underlies intentions to participate in actions on its behalf. A focus on extremist beliefs is important because theoretical models lend them considerable weight to precipitating violent extremism [27, 28]. We then test this tripartite model among a representative sample of 802 male prisoners in Texas who were reinterviewed around 10 months after release from prison. These data provide a means to assess levels of activism and radicalization among prisoners at a critical stage: as they are leaving prison and attempting to reintegrate into the community, and when they are most likely to bring beliefs and experiences from the prison back to the street.

### Theoretical framing of the imprisonment-extremism nexus

The origins of “inmate society,” including the structure of relationships and cultural values of prisoners, and its associated consequences, has been the subject of theoretical and empirical debate for decades [29]. One perspective, deprivation, is that prison institutions bring about functional adaptations to the loss of autonomy, liberty, safety, and relationships experienced while imprisoned [2, 30]. Another perspective, importation, instead emphasizes that behaviors and cultures within prisons are a reflection of the types of people who become imprisoned [31, 32]. More recently, scholars have turned their attention to how the prison experiences of formerly incarcerated persons influence their transition to the community upon their reentry to society. This is known as the exportation hypothesis [14]. We examine this critical stage (transition from prison to society) in the nexus between imprisonment and extremism because intentions to do harm in the name of a cause are somewhat (though not totally) constrained by the parameters of total institutions. Incarcerated people remain under near-constant surveillance and regulation, and the expression of activist and radical intentions may result in the transfer to a different prison unit or placement in solitary confinement [25, 33]. Yet, it is in the release to the community that the greatest potential for harm can be realized through acts of radicalized behavior. After all, over 90% of people incarcerated eventually leave [34]. We highlight three possible relationships between release from prison and activism/radicalization: (a) the extremism-continuity model, (b) the extremism-contraction model, and (c) the extremism-enhancement model. There are sound reasons to anticipate that these models capture the imprisonment-extremism nexus.

The *extremism-continuity* model holds that the transition from prison to the community is unrelated to activist and radical beliefs. It has long been argued that the beliefs of prisoners, often referred to as the convict or inmate code, are so strongly engrained that they accompany them upon release into the community. There is considerable research on the “convict code” documenting its pervasive character within prison as well as its overlap with the “code of the street” [7, 35–37]. The persistence of these beliefs as a cultural code that guides behavior even after release from prison is supported by arguments that prison culture is exported to the street [14]. Prison norms, behavior, and associations are translated to street settings, underscoring the role of imprisonment for life after release, including activism and radicalism that originated in prison. From this perspective, the empirical prediction is that leaving prison will not correspond with changes in activism or radicalism, and the imprisonment-extremism nexus may exist, but will not worsen or improve with reentry. Extremist orientations after prison are a mirror image of the extremist tendencies embraced while in prison.

The *extremism-contraction* model, in turn, shifts the focus from radicalization experiences inside of prison by emphasizing the realities of reentry. This model anticipates that activist and radical beliefs will decline upon leaving prison and returning to the community. Reentry is rife with challenges and demands that may require the expression or investment in extremist beliefs to take a backseat to more pressing needs. Examples of these needs include finding housing, reestablishing formal identity (social security cards, driver’s licenses, other licensing), rebuilding personal relationships with family and friends, and finding employment [13, 38]. These challenges are time-consuming for individuals who have been in prison for even short periods of time, much less sustained periods. The strength of values adopted in prison (e.g. activism and radicalism) is likely to wane without continued reinforcement by peers. Unlike prison, where individuals have idle time to socialize with fellow prisoners (e.g., recreational time, partake in religious activities, and interacting with others in prison work programs), the routine activities of reentry may limit access to anti-social peers, religious zealots, and ideas generative of political and social cynicism. The empirical prediction from the contraction model is that levels of activism and radicalism will decline upon release from prison.

The *extremism-enhancement* model takes the position that support for activism and radicalism increases upon release from prison. This process occurs because reentry experiences fail to meet the expectations on the part of those released from prison [39, 40]. It is well understood that prisoners are hopeful for a successful reentry [41]. Yet, those expectations often meet the harsh reality of reentry, even during periods of economic expansion and a tight labor market. The pace of societal change, particularly in the areas of technology, mobility, and employment, makes old skills and relationships less useful in finding and succeeding in work [42]. The roadblocks posed by a lack of technical skills may produce disillusionment with life on the outside. Former prisoners often must mend relationships with family and friends, who have progressed without the help of spouses, parents, or partners. The anomic state experienced by many such people is a consequence of the inability to master the changing realities of work, family, housing, community, and emerging technologies simultaneously. A response to this disillusionment may be the return to and/or extension of values and relationships, particularly activism and radicalism, learned inside prison, which presents a familiar and known pattern of values and relationships in an uncertain world. The empirical prediction from the enhancement model is that levels of activism and radicalism will exceed what is observed on the inside.

### Prior research on imprisonment and extremism

Evidence of a link between serving time in prison and the development of radicalized beliefs finds its strongest support among criminal justice officials and the media. For example, the U.S. Bureau of Prisons identifies “Security Threat Group” members as individuals who hold radicalized beliefs, particularly beliefs against the United States and state governments. Hamm found evidence of a link between gangs and extremist activities in U.S. prisons [43]. These groups can be traditional prison gangs, or groups that espouse race-supremacy, religious extremism, or some combination of ideologies. In the British context, Liebling and Straub interviewed 150 prisoners and 194 staff members at HMP Whitemore [44]. Staff members expressed fears that prisoners were becoming radicalized and were members of well-organized formal groups that had considerable power inside and outside the prison. In contrast, Jones conducted several case studies examining the role of imprisonment in the social process of becoming a terrorist in the United States, United Kingdom, Australia, the Philippines, Indonesia, and Pakistan [45]. He concluded that the evidence did not exist to support the view that radicalization was a consequence of time spent in prison. The case of Canada is interesting in this regard as Haggerty and Bucerius note that few researchers have been given access to interview prisoners in Canadian prisons [27], yet there are claims of a “threat of Islamist prison radicalization in Canada” [24].

De Vito underscores the importance of the relationship between the “inside” and the “outside” of prison in understanding both radicalization and de-radicalization [46]. This reflects the point made by Atran and colleagues regarding the need for a broader understanding of the social contexts of the development of activism and radicalization in relationship to involvement in terrorism [47]. Clearly, the harsh conditions of imprisonment (deprivation of liberty, regimentation of daily life, and isolation from pro-social activities and peers) play a role in fostering conditions where activism and radicalization may flourish. Despite its significance, there are relatively few studies of activism and radicalization in prison, and many such studies depend on the views of correctional employees rather than prisoners themselves. In large part, this is due to the difficulty in gaining access to prisoners and tracking them upon release [48, 49]. In a comprehensive review of the prevalence of Islam in U.S. prisons, Hamm notes that while there has been a dramatic increase in conversions to Islam among U.S. prisoners, it is hard to determine whether such conversions have led to increased or decreased terror activity, given the interventions to reduce such activity [50].

Perhaps the best evidence to date supporting the imprisonment-extremism nexus comes from the work of LaFree, Jiang, and Porter [21]. They used data from the Profiles of Individual Radicalization in the United States (PIRUS) to examine violent political extremism among 675 adults over the age of 21 who espoused far left, far right, Islamist, or single-issue ideologies. Their data were based on “open-source” records, such as newspaper articles, memoirs, and government documents. They made two sets of comparisons: (a) domestic extremists who spent time prison versus those who did not, and (b) domestic extremists who were radicalized while incarcerated versus those who did not. On the whole, their results provide support for the imprisonment-extremism nexus as they conclude that individuals who spent time in prison and were radicalized in prison were more likely to commit politically-motivated violent acts of terror relative to those who did not after release from prison.

One of the challenges in research on activism and radicalization is the lack of validated measurement tools [51]. An exception is the work of McCauley and Moskalenko [26, 52, 53] who developed and tested separate scales, known collectively as the Activism and Radicalism Intentions Scale (ARIS). The scale properties of these measures have been documented with samples comprised of different ages, nationalities, and social statuses, as well as with prisoners [54]. Moskalenko and McCauley argue that radicalism is both qualitatively and quantitatively different from activism and reflects a deeper commitment to action. Activism and radicalism also have different correlates and measure different concepts, leading the researchers to conclude that radicalism is not “simply” a higher score on the scale than activism and that the transition from activism to radicalization required more than a progression in the intensity of feelings toward a political cause. Certainly, the availability of validated scales provides a foundation for a more robust examination of the imprisonment-extremist nexus. Another major shortcoming of prior work in this area is its dependence on staff views of the issues, rather than measuring attitudes and behavior of individuals who have been released from prison. In addition, the design of earlier studies has been cross-sectional, making it difficult to identify the impact of prison while controlling for other factors, much less continuity and change.

### The current study

The purpose of this study is to examine continuity and change in activism and radicalism intentions among a sample of 802 prisoners in Texas interviewed several days before their release and reinterviewed around ten months after returning to the community. Texas is an ideal location to conduct this research, as the state has among the highest rates of incarceration in the United States with roughly 550 prisoners per 100,000 citizens. The Activism and Radicalism Intentions Scale was administered to all members of the sample at the baseline and post-release interviews. Using these data, we address two research questions:

1. Do activism and radicalism intentions change upon release from prison into the community?
2. What are the correlates of change in activism and radicalism intentions after release from prison?

A host of demographic, group membership, attitudinal, and behavioral measures were also collected as part of the interviews and are used to examine the correlates of continuity and change. To the best of our knowledge, this represents the first longitudinal assessment of activism and radicalism intentions upon release from prison.

## Methods

### Data

The data used to examine activism and radicalism during and after prison are from the LoneStar Project, or the Texas Study of Trajectories, Associations, and Reentry [55]. The research design involved interviewing prisoners just prior to their release and re-interviewing them upon their return to the community. Prisoners scheduled for release from the largest release unit in Texas constituted the sampling frame. A weekly list of scheduled releases was provided by the prison executive services office that included indicators relevant to the study purpose. Disproportionate stratified random sampling was used to generate a sample of 802 male respondents, drawn from the population of 15,644 prisoners released during the period of data collection. Sampling fractions differed by official gang classification, where people with non-zero levels of gang affiliation were oversampled by a factor of five. Sampling weights are used and denoted. The findings reported herein are representative of prisoners scheduled for release from prison in Texas during the study period.

Study protocols were approved by the Institutional Review Board at Arizona State University (STUDY00001971). Participation in the research was voluntary. Informed consent was obtained from those who chose to participate in the study. The rate of response, or number of respondents who completed an interview divided by the number of people eligible for enrollment, was 61%. The rate of cooperation, or the number of respondents who completed an interview divided by the number of people contacted, was 94%. No benefits were provided to respondents while incarcerated; however, monetary incentives (Walmart gift cards) were offered to respondents for post-release interviews.

Baseline data collection occurred between April and December 2016 in two prison units. The first was the release unit where our respondents were transferred prior to leaving custody. Prisoners who were designated as general population custody level were interviewed at this unit and constituted 95% of the sample. The remaining 5% of the sample was interviewed at the administrative segregation facility of the second unit, where high-custody level prisoners were housed. All these respondents were released from the aforementioned release unit. Trained interviewers affiliated with universities associated with the LoneStar Project employed computer assisted personal interviewing (CAPI) in both settings. Respondents were interviewed around two days prior to their release from prison. The mean duration of the interview was 110 minutes and item refusal/do not know rates were very low (<1%). A complete description of baseline data collection procedures is provided by Mitchell and colleagues [56].

Follow up data collection began May 2016 and was completed February 2018. Respondents were targeted for interviews one and ten months after release from prison, which constitute the wave 2 and 3 interviews. The geographic distribution of former prisoners across Texas required computer assisted telephone interviewing to conduct follow-up interviews, although we conducted face-to-face interviews across the state if respondents were incarcerated at their interview date. Follow-up interviews were facilitated using an extensive contact card generated from the in-prison interview and a client relationship management database to organize reminders, appointments, calls, and notes. The retention rates for these interviews were 66.3% (*N* = 532) and 64.1% (*N* = 514), consistent with large-scale studies of reentry [48, 49]. Transient lifestyles, legal cynicism, and limited support systems make former prisoners a hard-to-reach population, and a complete description of post-release collection procedures is provided by Fahmy and colleagues [48].

We examine change in activism and radicalism intentions between the baseline and wave 3 interviews. Ten months affords enough time between imprisonment and reentry to observe change in extremist orientations if it should exist. It is not possible to distinguish between immediate and distal temporal variation in extremism intentions because the scale was not administered during the wave 2 interview owing to study priorities. Missing data due to item nonresponse and a programming error applied to a small (*N* = 16) group of respondents led us to employ multiple imputation with chained equation for all analyses involving null hypothesis significance testing. However, all descriptive statistics are reported without imputation.

Our analytic sample consists of 514 respondents who completed baseline and wave 3 interviews. While our attrition rate is on par with prisoner reentry studies, it raises concerns of biased results, as respondents who were retained may differ from those who attrited. We have demonstrated in prior work that age, incarceration length, baseline honesty, and study commitment were positively associated with wave 3 retention, while gang membership, rearrest, and extrinsic motivations for study participation were negatively associated with retention [57]. We supplement those results by comparing retained (*N* = 514) and attrited (*N* = 288) respondents across the baseline measures derived from the activism and radicalism intentions scale (including reference group and item measures, described below). Only 2 of the 21 measures differed statistically, roughly what would be expected with a Type 1 error rate of 5%. These differences were observed for the importance of “other” groups (attriters score higher) and selecting religious as the most important group (attriters score lower). None of the 10 ARIS items differed.

### Dependent variables

The Activism and Radicalism Intentions Scale (ARIS) was administered to our respondents at the baseline and wave 3 interviews. The first step in the administration of the scale involves asking respondents to rank the importance of salient groups, including *country*, *racial/ethnic*, *religious*, or *other group*. We asked our respondents if there were “any other groups that you feel close to, such as a political group or organization,” and, if so, the name of the group. None of the respondents identified “family” (beyond Latino gang families) or “political” (beyond occasional mentions of Libertarianism or Presidential candidate/elect Trump) in the open-ended responses. The importance of these groups was rated on a scale ranging from 1 (“not at all important) to 7 (“extremely important”). The language of the questionnaire also noted that responding with a 4 was equivalent to a neutral position.

Respondents were then instructed to identify the group that was most important. The groups identified as the most important served as the referent for 10 questions about activism and radicalism intentions. At the baseline and wave 3 interviews, 15% and 25% of respondents indicated that there were no groups that were “most important” to them, respectively, which prevented us from analyzing continuity/change in the ARIS items (although we could still examine group importance). Nonetheless, about two-thirds of the post-release sample (*N* = 342) identified groups that were important to them at baseline and wave 3 interviews, which are subject to analysis.

Moskalenko and McCauley differentiate between legal and illegal forms of activism. Four of the items pertain to the former and focus on legal activities such as belonging, donating, volunteering, and protesting with groups. These items combine as the *activism intention scale* (AIS). Six of the items pertain to the latter and focus on illegal activities such as violence and outgroup retaliation. These items combine as the *radicalism intention scale* (RIS). The likelihood of engaging in these activities was rated on a scale ranging from 1 (“completely disagree”) to 7 (“completely agree”), and included 4 as a neutral response. A complete listing of the items can be found in the results section. Using a latent variable framework, a pooled cross-sectional confirmatory factor analysis led us to a modified two-factor model, consistent with prior research [26, 54]. The model exhibited acceptable psychometric properties (factor loadings>0.53; RMSEA 90% CI 0.04–0.07; CFI = 0.98; TLI = 0.97; AIS Cronbach’s α = 0.85; RIS α = 0.84). AIS and RIS factor scores were standardized across waves and constitute the dependent variable for the respondents who indicated a group was most important to them at the respective interviews.

### Independent variables

We examine the correlates of AIS and RIS after release from prison across a series of demographic, in-prison, and post-release measures, as well as ARIS reference groups (described above). *Age* is measured in years at the time of the baseline interview derived from dates of birth in prison records. Race and ethnicity included the most prevalent groups in the prison system. Four non-mutually exclusive and dummy coded (0 = no, 1 = yes) measures of race/ethnicity derived from self-reports include *Black*, *Latino*, *white*, and *mixed/other*. *Foreign born* is a self-reported indicator of whether the study subject was born in the United States (1 = yes, 0 = no). *Father* is an indicator of whether the respondent reported he had a child (1 = yes, 0 = no). *Education* was measured by the self-reported last grade of school completed based on seven response categories: less than sixth grade, junior high, incomplete high school, high school graduate/equivalency, incomplete college or specialized training, 4-year college graduate, and graduate professional training. These categories were rescaled to reflect years of educational attainment (e.g., high school graduate or equivalent = 12 years).

Several baseline (pre-release) measures of in-prison attitudes and experiences are included. *Gang affiliation* was determined by the prison system (1 = yes, 0 = no) [58]. *Low self-control* is derived from agreement with 13 items from the Brief Self-Control Scale [59], such as “You do certain things that are bad for you if they are fun.” Responses ranged from 0 “not at all like you” to 4 “very much like you” and averaged across the items, where higher scores equal poorer self-control (α = 0.80). *Legal cynicism* refers to views toward the law [60], and the construct consists of nine items, such as “People in power use the law to try and control people like you.” Response categories ranged from 0 “strongly disagree” to 3 “strongly agree,” and were averaged (α = 0.72). *Social support* is based on six items from the Multidimensional Scale of Perceived Social Support [61]. Based on a 4-point scale of agreement, the scale contains three measures of familial support (decision-making, general help, and emotional support) and peer support (e.g., sharing feelings, trust, talking), and the average was taken (α = 0.82). *Spirituality* is a scale derived from the survey instrument used to evaluate the Serious and Violent Offender Reentry Initiative (SVORI). Six items consisted of a 4-point scale of agreement and were averaged, and capture an internalized set of beliefs in a higher power, such as “You feel guided by your God in the midst of daily activities,” and manifestations of the traditions and formal practice of a faith, such as “You pray or meditate regularly” (α = 0.92). *Prison misconduct* is a variety score composed of 14 types of self-reported misconduct incidents during the current incarceration spell, ranging from less serious acts, such as “Have you refused to obey an order given by a member of the prison staff?,” to more serious acts, such as “Have you attacked a correctional officer with a weapon?” The item was standardized by years of incarceration. Measures from the Texas Departments of Public Safety (DPS) and Criminal Justice (TDCJ) include an indicator of cognitive ability, *IQ*, derived from the administration of the Wechsler Adult Intelligence Scale-Revised during prison classification, a count of *prior arrests* which was top-coded to the 99th percentile, the number of *years incarcerated*, and a count of the number of *prison terms* served in state facilities.

We examine whether several important characteristics of successful reentry were related to post-release AIS and RIS, consistent with our theoretical models where positive arrangements should be linked to reductions in extremism, while negative arrangements should be linked to increases in extremism. *In a relationship* measures self-reports of whether respondents were currently married or in a romantic partnership (1 = yes, 0 = no). *Employed* is a self-reported indicator of whether respondents supported themselves financially via a “legal job where income is taxed” (1 = yes, 0 = no). *Stable housing* indicates whether respondents lived in the same residence since release from prison (1 = yes, 0 = no). *Lives in a good neighborhood* is a measure of whether respondents agree with the statement that “You think your neighborhood is a good place to live” (1 = yes, 0 = no). Two measures of recidivism were included. *Rearrested* is based on DPS records of whether respondents were arrested for a new offense prior to their wave 3 interview (1 = yes, 0 = no). *Reincarcerated* is based on TDCJ records of whether respondents reentered state jail or prison facilities prior to the wave 3 interview (1 = yes, 0 = no). The final measure, *post-release time*, is the number of months between the baseline and wave 3 interviews.

#### Analytic strategy

We examine the relationship between reentry and extremism in two stages. First, we use multi-level modeling to test for intra-individual change in activism and radicalism upon release from prison. Two waves of data are nested within respondents, requiring a correction for non-independence across person-waves. Random intercepts give each respondent his own constant, that is, ARIS values at the baseline interview. A dummy measure of time, fixed to 0 at baseline and 1 at post-release interviews, captures continuity/change in the outcomes after release from prison. A random coefficient for time in the ARIS models allows the effect to vary across respondents. Coefficients that differ from 0 and are positive indicate increases in the outcomes, consistent with the extremism-enhancement model. Coefficients that differ from 0 and are negative signify reductions, consistent with the extremism-contraction model. Null coefficients indicate that post-release activism and radicalism intentions are equivalent to observations in prison, consistent with the extremism-continuity model.

Second, we estimate a series of bivariable and multivariable OLS regression models predicting post-release AIS and RIS scores. The former involves the estimation of a separate model for reach of our demographic, reference group, in-prison, and post-release covariates. The latter estimates two models with all of the aforementioned covariates predicting AIS and RIS scores simultaneously. The multivariable models control for baseline AIS and RIS scores, which allow the remaining coefficients for the remaining covariates to be interpreted as accounting for post-release change (the results generated from predicting change scores are substantively similar). Thus, these results identify the correlates of changes in extremist orientations among respondents with the transition back to the community.

The outcomes are standardized, as well as all non-binary independent variables, which allows for the interpretation of the coefficients as standard deviation unit differences in AIS and RIS, respectively. All stages of analysis involve weighting to correct for oversampling, report robust standard errors, are multiply imputed with chained equations using 10 datasets, and effect sizes are reported as Cohen’s *d* or *h* or standard deviation differences.

## Results

### Descriptive statistics of the sample

Table 1 displays the descriptive statistics for the non-ARIS study variables. The mean age of respondents was 41 years; the youngest respondent was 19 years and the oldest was 73 years.

**Table 1:** Descriptive statistics for the study variables.

	Full Sample				
	Valid *N*	Mean or %	SD	Min	Max
Demographics					
Age	514	41.03	(12.28)	19.60	73.26
Latino	512	32.83%	---	0	1
White	512	38.27%	---	0	1
Black	512	33.05%	---	0	1
Other	512	9.91%	---	0	1
Foreign born	513	2.72%	---	0	1
Father	514	66.49%	---	0	1
Education (in years)	513	11.71	(1.92)	6	18
In-Prison Measures					
Gang	514	10.07%	---	0	1
Low self-control	514	1.36	(0.74)	0	4
Legal cynicism	514	1.18	(0.43)	0	3
Social support	509	2.15	(0.67)	0	3
Spirituality	513	3.09	(0.90)	0	4
IQ	509	93.31	(13.09)	54	131
Prison misconduct	514	1.08	(3.75)	0	45.66
Prior arrests	514	8.44	(5.82)	0	35
Years incarcerated	514	4.97	(5.87)	0.04	34.98
Prison terms	514	2.23	(1.73)	1	14
Post-Release Measures					
In a relationship	508	23.07%	---	0	1
Employed	498	65.09%	---	0	1
Stable housing	503	66.19%	---	0	1
Lives in good neighborhood	514	46.35%	---	0	1
Rearrested	514	17.31%	---	0	1
Reincarcerated	514	8.01%	---	0	1
Post-release time (in months)	514	10.80	(2.62)	6.11	20.14

*Abbreviations*: *SD =* standard deviation; Min = minimum value; Max = maximum value.

Race and ethnicity was rather evenly distributed across Black (33%), Latino (33%), and white (38%) respondents, while 3% of respondents were born outside of the United States. Two-thirds of respondents were fathers at the time of the survey. Consistent with prior research on incarcerated individuals, the mean number of years of education fell short of completing high school (*M* = 11.7; *SD* = 2.0). Ten percent of respondents were designated as maintaining a gang affiliation. Prior to their release, respondents spent an average of five years incarcerated in state prison (*SD =* 5.9) and the average number of prison terms exceeded two (*SD* = 1.7).

Shifting to the post-release characteristics of the sample, under half of the respondents (41%) were in a romantic relationship when we interviewed them upon returning to the community. Two-thirds indicated they had a legal job with taxable income and maintain stable housing since release from prison. Under half (46%) claimed that they lived in a good neighborhood. In terms of recidivism, 17% and 8% of respondents had been rearrested or reincarcerated, respectively, prior to the date of the wave 3 interview, according to official records. Our wave 3 interview occurred under 11 months after release prison, although there was a fair amount of variation (*SD* = 2.6) across respondents.

### Intra-individual changes in activism and radicalism intentions

Table 2 presents the findings of a comparison of how respondents rated groups during and after imprisonment. The groups identified in prison became less salient 10 months into the reentry process. Respondents reported that country, racial/ethnic, and religious groups were about one-tenth of a standard deviation less important at the follow-up interview than in prison. This is consistent with the hypothesis that reentry is highly consuming of the lives of formerly incarcerated persons. If respondents are less likely to view these groups as important, they are less likely to endorse activist and radical views in support of them.

**Table 2:** Differences in ARIS reference groups pre- and post-release from prison.

		In-Prison		Post-Release			
	*N* =	514		514			
		Mean	(SD)	Mean	(SD)	*d*	| *t* |
Rank the importance of the following groups:	Country	5.26	(1.17)	5.12	(1.39)	-0.11	[2.00]
	Race/ethnic	4.82	(1.42)	4.64	(1.59)	-0.11	[1.87]
	Religious	4.75	(1.85)	4.52	(2.01)	-0.12	[2.38]
	Other	5.15	(0.88)	5.21	(1.25)	0.07	[0.49]
		%		%		*h*	| *t* |
Which group is the most important:	Country	26.8%		26.7%		-0.002	[0.09]
	Race/ethnic	14.3%		11.3%		-0.09	[1.46]
	Religious	42.7%		36.4%		-0.13	[2.08]
	Other	1.9%		0.7%		-0.11	[1.29]
	None	14.3%		24.9%		0.27	[3.87]

*Note*: Descriptive statistics are reported without imputation. Statistical significance (*t* statistics) was determined using a hierarchical (two-level) OLS model with waves nested within persons, where post-release wave (reference category = pre-release wave) was regressed on group items, based on multiple imputation with chained equations (*m* = 10 datasets).

*Abbreviations*: SD = standard deviation; *d* = Cohen’s *d*; *h* = Cohen’s *h*; *t* = *t* statistic (absolute value).

There were also changes in the groups that respondents deemed most important. The most notable finding was the overall increase in former prisoners indicating that “none” of these groups were important to them. Whereas 14% of respondents told us this at the baseline in-prison interview, 25% of them revealed this at the wave 3 interview. There were slight reductions in the endorsement of all groups, although respondents were statistically just as likely to select country, race/ethnicity, and other groups. Therefore, the gain in “no groups” category appeared to come primarily from those who endorsed religious groups while incarcerated, a decline of around 6 percentage points, despite religious group remaining the modal category at the post-release interview. To the extent that religious groups promote activism and radicalization, the declines observed in these measures support the view that religious values fall upon release from prison. These declines may cut “both ways” in that religion may play a role in enhancing such activism and radicalization but may also play a role in mitigating the deleterious effects of such beliefs [17]. These groups constitute the reference group that respondents use when responding to statement on activism and radicalism intentions.

Table 3 presents the results of intra-individual changes in the respective AIS and RIS scales and the items used to generate the scales. The overarching finding is that activism drops upon release from prison whereas radicalism essentially remains the same. All the items in the AIS fell in the post-release interview, by anywhere from 0.21 to 0.30 standard deviations. There was an overall 0.30 standard deviation reduction in the AIS; this supports the hypothesis that reentry weakens activism intentions. In contrast, the RIS reduced by 0.08 standard deviations but the effect size was statistically indistinguishable from zero (*p* = 0.28). The remaining items could not be distinguished statistically from zero. It is important to note that levels of activism far exceed radicalism; the mean response for the former was a “neutral” position, yet the latter was a “moderately disagree” position. Both constructs, and their associated items, maintained a fair amount of variation around the mean.

**Table 3:** Differences in ARIS items and scales pre- and post-release from prison.

		In-Prison		Post-Release			
		Mean	(SD)	Mean	(SD)	*d*	| *z* |
**Activism Intention Scale (AIS)**		**0.19**	**(0.97)**	**-0.15**	**(1.01)**	**-0.30**	[4.10]*
1	Join or belong to an organization that fights for [GROUP] rights	4.42	(1.97)	3.96	(2.11)	-0.21	[2.83]*
2	Donate money to an organization that fights for [GROUP] rights	4.56	(1.84)	3.98	(2.06)	-0.30	[3.70]*
3	Volunteer time working for an organization that fights for [GROUP] rights	4.24	(1.95)	3.70	(2.06)	-0.26	[3.37]*
4	Travel for one hour to join in a public rally, protest, or demonstration in support of [GROUP]	3.98	(2.05)	3.47	(2.27)	-0.22	[2.79]*
**Radicalism Intention Scale (RIS)**		**0.02**	**(0.97)**	**-0.04**	**(1.02)**	**-0.08**	**[1.09]**
5	Support an organization that fights for [GROUP] rights even if it sometimes breaks the law	1.80	(1.92)	1.40	(1.84)	-0.17	[2.24]*
6	Support an organization that fights for [GROUP] rights even if it sometimes resorts to violence	1.31	(1.78)	1.35	(2.00)	0.06	[0.83]
7	Participate in a public protest against oppression of [GROUP] even if you thought it might turn violent	1.90	(2.09)	1.67	(2.09)	-0.10	[1.33]
8	Attack police or security forces if you saw them beating members of [GROUP]	1.58	(1.94)	1.80	(2.24)	0.12	[1.69]

*Note*: Descriptive statistics are reported without imputation and only for respondents who provided an ARIS reference group (non-imputed person *N* range: 326–340). Point estimates were generated from multiply imputed (*m* = 10, person *N* = 342) two-level OLS models where waves are nested within persons, regressing a binary indicator of post-release wave (reference category = pre-release wave) on standardized activism and radicalism intentions items and scales.

* *p <* .05.

*Abbreviations*: SD = standard deviation; *d* = Cohen’s *d*. *z* = *z* statistic; [GROUP] = ARIS reference group.

The models predicting intra-individual change in AIS and RIS also included a random coefficient for wave, which was coded 0 at the baseline interview and 1 at the follow-up interview. The random coefficients for wave in non-imputed models (*N* = 327, N*T = 654) contained standard deviations of 0.97 and 0.78 for the AIS and RIS models, respectively, which suggests that there is substantial variability across respondents in changes in activism and radicalism intention. We now move to the question of what explains those changes.

### Bivariable correlates of activism and radicalism intentions

The bivariable analyses are reported in Table 4. We find little evidence in support of demographics as correlates of ARIS. Only age was negatively associated with radicalism levels, a standardized drop of 0.20 units. No other measures in the demographic domain were associated with post-release AIS and RIS scores. In terms of the ARIS reference groups selected by respondents, none were unrelated to post-release AIS scores, while three were related to RIS scores. Those who selected race/ethnicity and other groups as the reference category scored 0.69 and 1.52 standard deviations higher in RIS than their comparison groups, respectively. In contrast, respondents who selected religious groups as their reference category scored 0.27 standard deviations lower in RIS. Reference groups appear to matter more for levels of radicalism than activism.

**Table 4:** Bivariable OLS regressions predicting post-release standardized levels of Activism Intention Scale (AIS) and Radicalism Intention Scale (RIS).

	AIS Post-Release Score			RIS Post-Release Score		
	*b*	(se)	| *t* |	*b*	(se)	| *t* |
Demographics						
Age ^a^	0.128	(0.070)	|1.84| †	-0.204	(0.071)	|2.89| *
Latino ^b^	-0.065	(0.142)	|0.46|	-0.053	(0.135)	|0.39|
White ^b^	0.171	(0.131)	|1.31|	-0.076	(0.133)	|0.57|
Black ^b^	0.015	(0.137)	|0.11|	0.035	(0.135)	|0.26|
Other ^b^	0.005	(0.216)	|0.02|	-0.156	(0.180)	|0.88|
Foreign born ^b^	-0.481	(0.423)	|1.14|	-0.027	(0.452)	|0.06|
Father ^b^	-0.123	(0.139)	|0.89|	-0.232	(0.147)	|1.58|
Education ^a^	-0.018	(0.072)	|0.25|	-0.094	(0.059)	|1.60|
ARIS Reference Category						
Country ^b^	0.212	(0.131)	|1.62|	-0.177	(0.127)	|1.40|
Race/ethnicity ^b^	-0.261	(0.187)	|1.39|	0.686	(0.231)	|2.97| *
Religion ^b^	-0.102	(0.130)	|0.79|	-0.272	(0.128)	|2.13| *
Other ^b^	0.606	(0.367)	|1.65|	1.517	(0.497)	|3.05| *
In-Prison Measures						
Baseline AIS ^a^	0.355	(0.071)	|4.98| *	0.104	(0.060)	|1.74| †
Baseline RIS ^a^	0.112	(0.066)	|1.70| †	0.465	(0.071)	|6.64| *
Gang ^b^	-0.196	(0.114)	|1.72| †	0.066	(0.115)	|0.57|
Low self-control ^a^	-0.028	(0.072)	|0.39|	0.224	(0.074)	|3.03| *
Legal cynicism ^a^	0.157	(0.062)	|2.51| *	0.249	(0.061)	|4.09| *
Social support ^a^	-0.015	(0.072)	|0.20|	-0.072	(0.063)	|1.15|
Spirituality ^a^	0.075	(0.073)	|1.02|	-0.087	(0.068)	|1.27|
IQ ^a^	-0.093	(0.063)	|1.48|	-0.108	(0.072)	|1.49|
Prison misconduct ^a^	-0.060	(0.063)	|0.97|	0.089	(0.048)	|1.86| *
Prior arrests ^a^	0.031	(0.057)	|0.54|	0.034	(0.062)	|0.54|
Years incarcerated ^a^	0.140	(0.059)	|2.38| *	-0.087	(0.064)	|1.35|
Prison terms ^a^	0.033	(0.056)	|0.59|	-0.019	(0.050)	|0.38|
Post-Release Measures						
In a relationship ^b^	-0.161	(0.153)	|1.05|	-0.151	(0.124)	|1.21|
Employed ^b^	-0.100	(0.139)	|0.71|	0.000	(0.141)	|0.00|
Stable housing ^b^	-0.229	(0.132)	|1.74| †	-0.154	(0.140)	|1.10|
Lives in good neighborhood ^b^	0.093	(0.131)	|0.71|	0.208	(0.131)	|1.59|
Rearrested ^b^	0.025	(0.163)	|0.15|	0.297	(0.190)	|1.56|
Reincarcerated ^b^	-0.390	(0.253)	|1.54|	0.090	(0.229)	|0.40|
Post-release time ^a^	0.006	(0.054)	|0.10|	0.085	(0.065)	|1.32|

^a^ *x* standardized

^b^ dichotomous

† *p* < .10

* *p* < .05.

*Abbreviations*: *b* = *y* standardized coefficient; (se) = robust standard error; [*t*] = *t* statistic.

The in-prison domain contained several statistically significant correlates of AIS and RIS. The baseline AIS and RIS scores represented the strongest predictors of the respective post-release outcomes. Although large, the standardized scores of 0.36 (*p* < .001) and 0.47 (*p* < .001) offer evidence of regression to the mean. Lower levels of self-control was associated with higher radicalism, but not activism. Legal cynicism was positively related to both AIS (0.16 standard deviations) and RIS (0.25 standard deviations). Respondents who spent longer spells in prison maintained higher levels of activism, where an additional six years in prison (relative to the mean of five years) corresponded with 0.14 standard deviations higher in AIS. Respondents who violated prison rules nearly five times per year maintained RIS scores that were 0.09 standard deviations higher than respondents who violated only one rule per year.

Contrary to our expectations, post-release scores on AIS and RIS levels were unrelated to measures capturing relationships, employment, neighborhood, recidivism, or temporal proximity to prison release.

### Multivariable correlates of activism and radicalism intentions

The results presented in Table 5 extend our findings by subjecting the imprisonment-extremist nexus to a multivariable analysis. By controlling for baseline levels of AIS and RIS, that is, extremist orientations in prison, our results are capturing the sources of post-release variation.

**Table 5:** Multivariable OLS regressions predicting post-release standardized levels in Activism Intention Scale (AIS) and Radicalism Intention Scale (RIS).

	AIS Post-Release Score			RIS Post-Release Score		
	*b*	(se)	| *t* |	*b*	(se)	| *t* |
Demographics						
Age ^a^	0.075	(0.086)	|0.87|	-0.046	(0.076)	|0.60|
Latino [ref: white only] ^b^	0.033	(0.148)	|0.22|	-0.026	(0.150)	|0.17|
Black [ref: white only] ^b^	-0.106	(0.165)	|0.64|	0.090	(0.146)	|0.62|
Other [ref: white only] ^b^	-0.043	(0.177)	|0.25|	-0.171	(0.137)	|1.25|
Foreign born ^b^	-0.080	(0.375)	|0.21|	-0.184	(0.412)	|0.45|
Father ^b^	-0.182	(0.130)	|1.40|	-0.157	(0.135)	|1.17|
Education ^a^	0.062	(0.071)	|0.87|	0.099	(0.060)	|1.65| †
ARIS Reference Category						
Country [ref: race] ^b^	0.334	(0.207)	|1.62|	-0.454	(0.199)	|2.28| *
Religion [ref: race] ^b^	0.014	(0.208)	|0.07|	-0.634	(0.204)	|3.11| *
Other [ref: race] ^b^	0.717	(0.561)	|1.28|	0.673	(0.473)	|1.42|
In-Prison Measures						
Baseline AIS ^a^	0.322	(0.080)	|4.04| *	-0.019	(0.067)	|0.28|
Baseline RIS ^a^	0.015	(0.076)	|0.20|	0.418	(0.071)	|5.86| *
Gang ^b^	-0.230	(0.124)	|1.86| †	0.075	(0.123)	|0.61|
Low self-control ^a^	-0.030	(0.065)	|0.46|	0.049	(0.066)	|0.74|
Legal cynicism ^a^	0.220	(0.069)	|3.19| *	0.163	(0.062)	|2.63| *
Social support ^a^	-0.005	(0.066)	|0.08|	0.005	(0.062)	|0.09|
Spirituality ^a^	0.035	(0.070)	|0.50|	0.098	(0.063)	|1.55|
IQ ^a^	-0.112	(0.062)	|1.81| †	-0.100	(0.068)	|1.46|
Prison misconduct ^a^	-0.040	(0.034)	|1.20|	-0.002	(0.032)	|0.06|
Prior arrests ^a^	0.024	(0.068)	|0.36|	0.042	(0.069)	|0.61|
Years incarcerated ^a^	0.053	(0.071)	|0.75|	-0.012	(0.060)	|0.20|
Prison terms ^a^	0.060	(0.066)	|0.90|	-0.036	(0.068)	|0.53|
Post-Release Measures						
In a relationship ^b^	-0.165	(0.150)	|1.10|	-0.172	(0.126)	|1.36|
Employed ^b^	0.142	(0.151)	|0.95|	0.166	(0.137)	|1.22|
Stable housing ^b^	-0.213	(0.154)	|1.38|	-0.008	(0.139)	|0.06|
Lives in good neighborhood ^b^	0.015	(0.134)	|0.11|	0.203	(0.124)	|1.64|
Rearrested ^b^	0.135	(0.183)	|0.73|	-0.091	(0.184)	|0.50|
Reincarcerated ^b^	-0.538	(0.270)	|2.01| *	-0.247	(0.210)	|1.18|
Post-release time ^a^	0.009	(0.056)	|0.16|	0.092	(0.067)	|1.39|
Constant	0.096	(0.294)	|0.33|	0.422	(0.271)	|1.56|

^a^ *x* standardized

^b^ dichotomous

† *p* < .10

* *p* < .05.

*Abbreviations*: [ref.] = reference category; *b* = *y* standardized coefficient; (se) = robust standard error; | *t* | = absolute *t* statistic.

In terms of activism, the results indicate that neither the demographic nor the ARIS reference groups were associated with changes in the AIS upon release from prison. We observed a 0.22 standardized increase associated with higher scores on legal cynicism. Similar to our bivariable results, our post-release measures did a poor job of accounting for variation in changes in activism intentions. Only reincarceration was linked to changes in the outcome, where we observed a 0.53 standard deviation reduction in activism intentions among the minority of our sample who recidivated to prison.

In terms of radicalism, our results identify very few correlates of post-release change in RIS scores. Respondents who indicate race/ethnicity as the most important group scored higher than those indicating country or religion (and statistically indistinguishable from “other category”). Among the in-prison measures, only legal cynicism was positively associated with post-release changes in radicalism intentions—a standardized coefficient of 0.16. None of the post-release measures could explain variation in the changes in radicalism intentions.

## Discussion

The specter of radicalization in prison has long concerned prison officials and the public. This issue takes on added significance owing to mass incarceration and reentry in the United States [38], leading us to focus on the exportation of extremist orientations from prison to the street in a longitudinal study of prisoner reentry in Texas. We introduced and tested a tripartite theoretical model with empirical predictions of positive (enhancement model), negative (contraction model), and null (continuity model) changes in activism and radicalism intentions upon release from prison. Overall, we found no evidence in support of enhancement and mixed evidence in support of contraction (for activism) and continuity (for radicalism). We arrive at three main conclusions that reflect on key aspects of the imprisonment-extremism nexus.

First, our results offer persuasive evidence of reductions in activism but not radicalism upon release from prison. Perhaps this difference emerges because activism requires a commitment of time and capital, whereas radicalism is more normative without the same time- or resource-intensive commitments. Put differently, violence is the expression of radicalized intentions, while activism is typically non-violent, but more time-intensive. It should be noted that this finding is consistent with the McCauley and Moskalenko contention that activism and radicalism are different concepts that measure different domains. We return to our tripartite theoretical models for guidance in assessing these findings. The contraction model best accounts for the changes observed in activism, perhaps reflecting the intensity of the reentry process in finding housing, work, and re-establishing social capital. As Western documented, the first few weeks and months post-release are busy times, critical to post-release success or failures [13]. Activism requires a commitment of time and social capital and maintaining the relationships necessary to sustain an activist set of beliefs presents considerable challenges. Fulfilling the commitment to conditions of parole, the workplace, and family are time consuming. This is particularly true of the first few months after release, as parolees need to be vigilant about such obligations early on in their release period, activities that consume more time and attention, leaving less available energy and capital for pursuing activist goals [6, 38, 62]. The absence of prison influences could facilitate turning away from extremism [63]. The fact that salient groups—race, country, religious, and other groups—fell in importance upon release from prison lends credence to this assertion.

In contrast, radicalism seems to be best accounted for by the continuity model, where in-prison beliefs extend beyond prison and into the community. In other words, there is no alteration in radicalism intentions with the transition from the prison to the street. Recall that base rates of radicalism in our sample were quite low, perhaps not allowing for much room to decline upon release from prison. That said, the base rates we observe are comparable to Moskalenko and McCauley’s findings among college students [26]. We should add that we find no evidence in support of the enhancement model—activism and radicalism intentions exhibit patterns of contraction or continuity, but do not increase with reentry to the community. The differences in activism and radicalism post-release underscore the importance of better measurement in explaining extremist behavior, both in the context of prison as well as post-release experiences. It is also important to note that there was significant variability across formerly incarcerated persons in their activist *and* radicalized beliefs. Understanding this variability is important to advancing policy, practice, and research on extremism.

This leads us to our second key point, which is that we observe few predictors of activism and radicalism intentions, albeit with one exception: legal cynicism. Legal cynicism refers to a set of cultural beliefs consistent with antipathy toward the law and its agents [64]. Manifestations of legal cynicism may range from failure to report crime to open opposition to the law, by which those tasked with its enforcement to follow scripts that support conflict resolution through non-legal or vigilante channels [65–68]. Legal cynicism is consistent with activism and radicalism intentions in that both represent opposition to the legitimacy of the law and legal authorities. There is nascent evidence of the linkages between collective strains and extremist orientations [69]. We believe that this is an important finding. With an average of five years spent in prison, the members of our sample experienced the impact of such a total institution. This underscores the power of institutional socialization, something evident in measures such as legal cynicism which was consistently associated with higher scores on the activism and radicalism scales. We lack direct measures that could account for this but suspect that years in prison (and the experiences leading up to prison of cycling through arrest, jail and imprisonment) produce disregard for and undermine respect for the criminal justice system. While not measured directly in the current paper, other research provides support for the links between procedural justice and legitimacy in prison and compliance in the context of American, British, Dutch, and Slovene prisons [70–73]. Such an anomic state impacts other domains, such as beliefs, and persists well beyond the time in prison and may well lead to activism and radicalism.

The importance of a racial group increased radicalism, highlighting the salience of group membership for the extremist process. In prison, racial identity is believed to be especially significant, something that both prisoners and prison staff help to construct [74, 75]. Because prisons strip individuals of their identity and in the process challenge individual identity, the significance of racial identification may be heightened, and lead to radicalization [76]. And as Jasko et al. note, loss of “significance” can be a powerful motivation for becoming radicalized [28]. It is seemingly paradoxical that leaving prison would result in elevating radicalism intentions among those who identify race as the most important social group. One would expect that the underlying pressures of the total institution should subside in the community [77, 78]. Yet racial distinctions could be heightened in the community where the racial order diverges from that of the prison. In other words, the rules of the institution no longer exist, and as a consequence could give rise to such radicalist intentions in the midst of adjustment to a new social order in the community. Thus, it is the intersection of racial identity and personal significance in prison that provide a potential explanation for the persistence of racial groups to radicalization. We would expect that, with longer-term follow-up, the racial residual of the prison would decline.

Third, it is surprising that none of our measures of post-release experiences were related to activism or radicalism intentions. While we observed sample-level reductions in activism intentions, as well as significant variation in post-release changes in activism and radicalism intentions, we have little to offer in the way of an empirically-supported explanation. This could be a measurement issue. After all, our post-release measures concentrate primarily on whether or not certain experiences took place rather than the quality of relationships, employment, or housing. Perhaps introducing greater variation on these measures, or capturing combinations of reentry experiences (e.g., relationship + employment), would better explain variation in activism and radicalism intentions, as there is sound theoretical rationale to anticipate that the mechanisms outlined in the contraction and enhancement models are reasonable explanations for the findings observed here. We are at a nascent stage in accounting for these outcomes, and more research and theory development are high on the agenda of testing the exportation hypothesis generally as well the explanation of intra-individual changes in activism and radicalism intentions.

Future research could address limitations found in our study. First, it is necessary to link attitudes to behavior. Even among prisoners, activism and radicalism *intentions* are low base rate phenomena, but studying such behaviors is important. These include verbal abuse of groups (e.g., demographic, religious), online expressions of extremism, including direct threats, and ideologically-based attempted or completed violence. The use of alternative measures of extremism orientations, such as the VERA-2 scale [79], should also be explored. Second, extending studies to samples culturally and geographically distinct from our own is an important next step. A more diverse sample, perhaps employing a multi-jurisdictional or country sampling frame, would be a welcome addition to such research [80]. While our sample is broadly representative of Texas prison releases, the state is not representative of the variation in prisoner characteristics nationwide, much less worldwide, as the United States continued to be an outlier in incarceration. Still, the issues of activism and radicalization extend, and are perhaps more salient, beyond the United States. The growth of corrections research and policy in the last two decades in Canada, the United Kingdom, and countries in the European Union offers possibilities for comparative work in this area [81]. Such advances would require considerable expenditures of money and effort. Finally, there is tremendous value in longitudinal study designs, but there is a need to examine the continuity/change in extremist orientations associated with entering and exiting prisons. Study designs that established baseline extremist orientations for those beginning a sentence could more accurately examine the impact of prison on the origins of activism and radicalism among inmates. Also, more post-prison follow-up periods would advance many lines of important research focusing on reentry. Indeed, it is possible that the reductions in activism we observe represent short-term alterations in a longer trajectory of activism intentions that subside with continued reentry and reintegration. Although we found no baseline differences in ARIS scores between respondents who were retained and those who attrited, it is possible that these respective groups maintain differential experiences post-release from prison. To the best of our knowledge, this study represents the first attempt to gather systematic information on extremist orientations from the same individuals interviewed during and after incarceration.

In conclusion, there has been considerable speculation that imprisonment is a corollary of radicalization yet little solid empirical evidence exists on the topic. This study aimed to contribute systematic analysis to this debate using longitudinal data, and the resulting findings provide broader reentry implications. The most important of these is that we find little in the way of exportation of beliefs or group affiliations from prison to the street. Put directly, what is learned and practiced in prison appears to quickly lose its vitality on the street. This can be seen most directly in the expression of support for religious beliefs and practices in prison that fades in under a year after release. The group processes that support values in prison no longer function after release; this can have positive and negative consequences, depending on the nature of the values. We suspect that this decline in prison allegiances and values is a consequence of the challenges of reentry. After being confined for many years, the transition to society is complex. Establishing contacts with employment, family, housing and the training required to function in a changed world are time-consuming activities. The sharper decline in activism may be a part of this pattern, as activism requires a commitment for activities related to a belief or cause. This suggests an important role for programming, perhaps in life or employment skills, that would work to keep released inmates active and involved in prosocial activities and out of groups with activist or radicalized beliefs. Reentry planning and programming have a key role to play in transitioning from prison to society. Part of that role includes strengthening prosocial relationships, beliefs, and activities upon release. Given our finding that activism intentions decline upon release, it is important to not impede the natural process of reentry that seems to suppress activism intentions, and enhance involvement in prosocial activities. While these are thought to be essential elements of a successful transition from prison, they may also play a role in reducing activism intentions. Certainly providing alternatives to groups that harbor or enhance such values may pay dividends in reducing post-release beliefs and activities of individuals who hold extremist beliefs.
